# Cellular expression and function of naturally occurring variants of the human ABCG2 multidrug transporter

**DOI:** 10.1007/s00018-019-03186-2

**Published:** 2019-06-28

**Authors:** Boglárka Zámbó, Orsolya Mózner, Zsuzsa Bartos, György Török, György Várady, Ágnes Telbisz, László Homolya, Tamás I. Orbán, Balázs Sarkadi

**Affiliations:** 1 Institute of Enzymology, Research Centre for Natural Sciences, Hungarian Academy of Sciences, Magyar Tudosok krt. 2, Budapest, 1117 Hungary; 2 Department of Biophysics and Radiation Biology, Semmelweis University, Tuzolto u. 37-47, Budapest, 1094 Hungary

**Keywords:** ABCG2, BCRP, MXR, Membrane transporter, Natural variants

## Abstract

The human ABCG2 multidrug transporter plays a crucial role in the absorption and excretion of xeno- and endobiotics; thus the relatively frequent polymorphic and mutant ABCG2 variants in the population may significantly alter disease conditions and pharmacological effects. Low-level or non-functional ABCG2 expression may increase individual drug toxicity, reduce cancer drug resistance, and result in hyperuricemia and gout. In the present work we have studied the cellular expression, trafficking, and function of nine naturally occurring polymorphic and mutant variants of ABCG2. A comprehensive analysis of the membrane localization, transport, and ATPase activity, as well as retention and degradation in intracellular compartments was performed. Among the examined variants, R147W and R383C showed expression and/or protein folding defects, indicating that they could indeed contribute to ABCG2 functional deficiency. These studies and the applied methods should significantly promote the exploration of the medical effects of these personal variants, promote potential therapies, and help to elucidate the specific role of the affected regions in the folding and function of the ABCG2 protein.

**Electronic supplementary material:**

The online version of this article (10.1007/s00018-019-03186-2) contains supplementary material, which is available to authorized users.

## Introduction

The human ABCG2 belongs to the ABC superfamily and is responsible for the transfer of wide variety of endogenous substances through the plasma membrane [1, 2]. ABCG2 is present in the GI tract [3, 4], the blood–brain barrier [5], the placenta [6], and in various stem cells [7]. This transporter protein is very important for the absorption, distribution, metabolism, and excretion of various pharmacological agents and the severity of their potential toxicity (ADME-Tox parameters, [8]). In addition to its crucial defense role in normal tissues, ABCG2 is also involved in multidrug resistance in cancer cells [9, 10].

There is growing evidence that the reduced level and/or function of ABCG2 contribute to the development of the painful disease, gout. Recent studies suggest that ABCG2 dysfunction causes a decrease in the uric acid excretion, especially in the intestine, resulting in hyperuricemia [11]. Genome-wide association studies (GWAS) revealed that one of the common single nucleotide polymorphisms (SNP), ABCG2-Q141K, is strongly associated with higher serum urate levels [12, 13] and gout [14–17]. It has also been established that numerous missense mutations in the *ABCG2* gene are also present in the human population [18–20], and some of these mutations may lead to reduced function and/or expression of the protein.

In our recent work, we found a new missense variant, ABCG2-M71V, which resulted in about 50% reduction in the expression of the ABCG2 protein in the erythrocyte membrane of heterozygous carriers [21]. This mutation was found to be relatively frequent in a heterozygous form (about 1%) in Europe, and we could demonstrate a major effect of this mutation on the expression and function of the ABCG2 protein. According to SNP databases (e.g. NCBI SNP database), several hundred other missense mutations may be present in the *ABCG2* gene in the general human population, while the molecular and cellular effects of most of these mutations are currently unexplored.

In 2017, several *ABCG2* gene mutations were found in Europe in a cohort of gout patients [22], and while the present manuscript was under review, the same group published a further analysis of these variants in clinical samples and performed experiments to assess the expression and function of these variants [23]. In the present work we have performed a comprehensive analysis of the expression, localization, and activity of the naturally occurring M71V, R147W, T153M, K360Δ, F373C, R383C, T434M, and S476P ABCG2 variants and compared their effects to the wild-type protein, as well as to the widely present (about 20% of heterozygotes in Europe) ABCG2-Q141K polymorphism, reducing both expression and function of ABCG2 [24–27]. For large-scale rapid screening of missense ABCG2 variants we present here an efficient transient expression system, while a detailed cellular localization and processing study was performed using stable cellular ABCG2 expression. Our cellular studies, along with the recently published atomic level model of the ABCG2 protein [28], appoint those regions which are essential for the proper folding, trafficking and functioning of this protein.

## Results

### Transient expression of the ABCG2 variants in human cell lines

In order to explore the effects of nine naturally occurring ABCG2 variants in human model cells, we performed a transient expression of these proteins in HEK cells. To ensure controlled, uniform transfection levels, we have generated plasmids which contained the cDNA of *ABCG2* and *EGFP* separated by an IRES sequence. Using these constructs, the successfully transfected cells could be separated by flow cytometry, based on EGFP fluorescence. Moreover, based on the EGFP levels, a similar efficiency of transfection could be used for cellular ABCG2 expression studies.

**Fig. 1:**
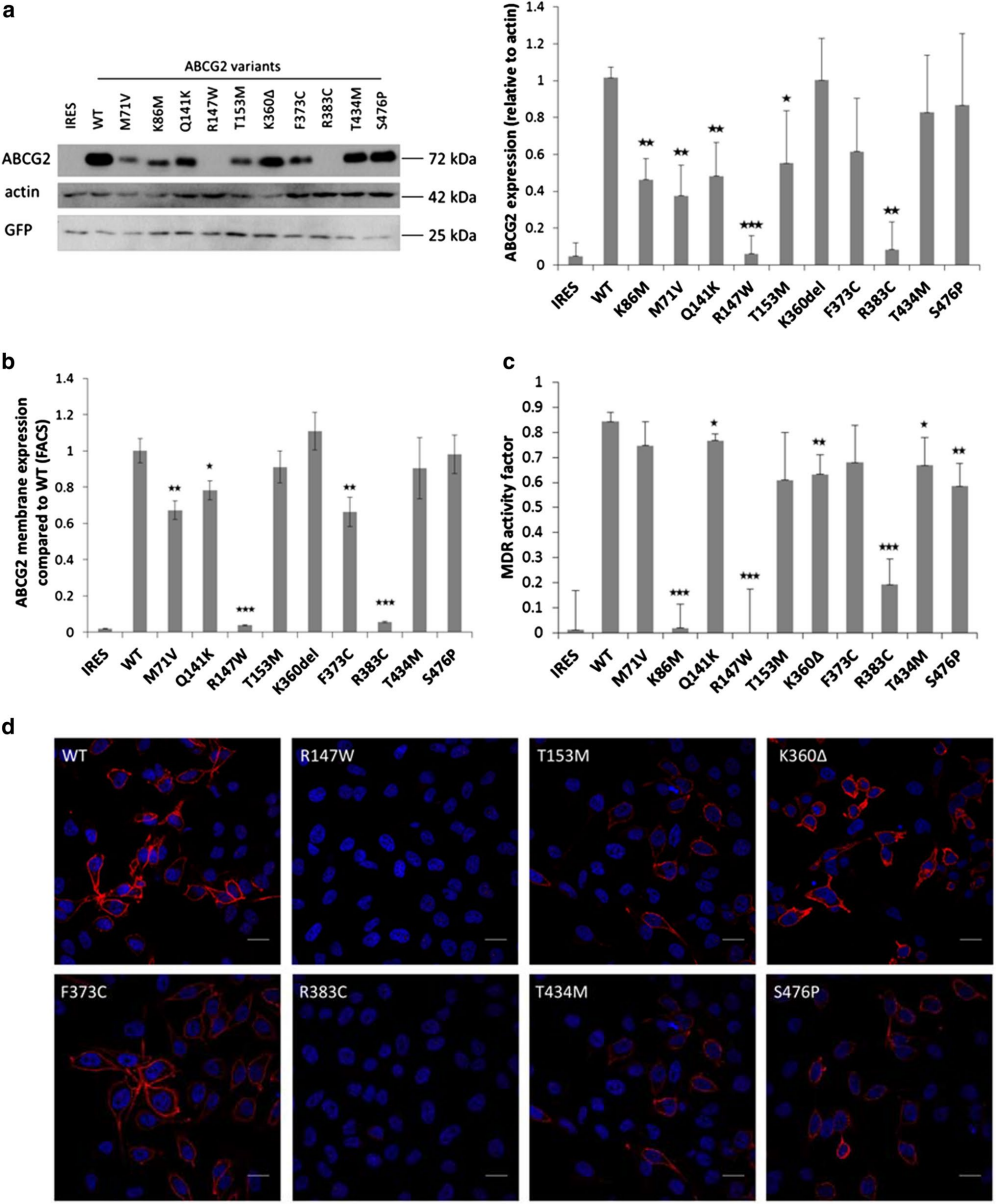
Transient expression and dye extrusion capacities of WT and mutant ABCG2 forms in HEK cells 48 h after transfection. **a** Western blot of transfected HEK cells. (Left panel: representative Western-blot, right panel: densitometry results of four blots.) The R147W and R383C variants showed no measurable expression, and the K86M, M71V and Q141K variants showed significantly reduced expression. The T153M and F373C variants showed slightly lower expression than WT, while K360Δ, T434M and S476P were similar to WT. **b** Determination of cell surface expression of ABCG2 by 5D3 antibody labelling. We could not detect surface expression in the case of the R147W and R383C variants; M71V and F373C showed significantly lower cell surface expression than WT (four independent parallel experiments). **c** Measurement of Hoechst dye extrusion capacity. In case of the R147W and R383C variants we could measure similar, very low MDR activity factors than in the case of the non-functional mutant K86M, and the IRES-empty vectors. The Q141K, K360Δ T434M and S476P proteins showed somewhat lower activities than the WT ABCG2 (four independent parallel experiments). **d** Confocal images of HEK cells transiently expressing the ABCG2 variants. The variants which could be expressed all reached the plasma membrane. The scale bars represent 20 μm. Blue: DAPI, red: ABCG2. The stars indicate the results of t-tests; *p < 0.05, **p < 0.01, ***p < 0.001

As documented in Fig. 1a, the ABCG2 protein expression in the HEK293 cells was examined by Western blotting. We found that the total expression levels of the K360Δ, F373C, T434M, and S476P ABCG2 variants were similar to that of the wild-type protein, the variants M71V, Q141K, and T153M had lower but well measurable expression, while the R147W and R383C variants showed no measurable expression in this system. As a well-studied mutant, the catalytically inactive K86M variant was also expressed in these studies, showing a low but well measurable expression level. Interestingly, in all cases when ABCG2 expression was well measurable, most of the transiently expressed protein was fully glycosylated.

In the experiments shown in Fig. 1b, the transiently transfected HEK293 cells were labeled with 5D3 monoclonal antibody, which recognizes an extracellular epitope of the ABCG2 protein, thus indicating the amount of ABCG2 present in the plasma membrane, as measured by flow cytometry after EGFP gating (see “Materials and methods”). The results showed that two of the mutants, R147W and R383C (in accordance with the total ABCG2 levels), could not be found in the plasma membrane. The mutant ABCG2 variants M71V, Q141K, and F373C showed significantly lower membrane expression levels than the wild type protein, while the plasma membrane expression of the T153M, K360Δ, T434M, and S476P variants was similar to the wild type.

Figure 1c demonstrates the transport activity measurements of the ABCG2 variants transiently expressed in HEK cells. The measurement of the Hoechst dye extrusion is a very sensitive assay, and the relatively high level of the ABCG2 variants expressed transiently can provide a high transport activity (MAF) value [29]. Thus, even in the case of a lower membrane expression level, most of the studied protein variants showed an efficient dye extrusion. Still, there was no measurable dye transport activity in the case of the R147W and R383C mutants, similar to the K86M inactive variant (or the empty vector, containing IRES-EGFP).

Figure 1d shows confocal microscopy images of the HEK cells transiently expressing the ABCG2 variants. While the Western blot or the surface expression values obtained by the 5D3 antibody binding data provide more quantitative data (see Fig. 1b), these images also indicate a corresponding expression level and localization of the ABCG2 protein. These images show that the T153M, K360Δ, F373C, T434M, S476P variants localized—at least partly—in the plasma membrane, while the expression of the R147W and R383C variants was negligible.

### Transient expression of the ABCG2 variants in Sf9 insect cells

**Fig. 2:**
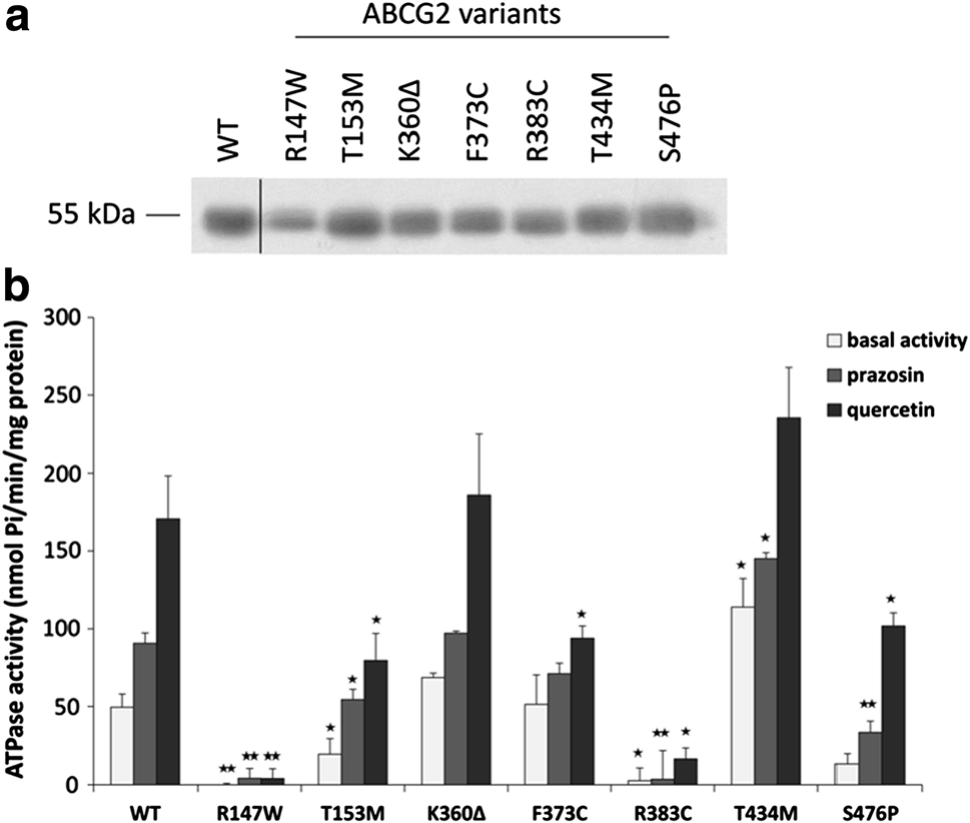
ABCG2-ATPase activity measurements in isolated Sf9 cell membranes containing the ABCG2 variants. **a** Western-blot analysis of isolated membranes. All ABCG2 variants could be expressed in these cells at similar levels as the WT protein. **b** Basal, prazosin- and quercetin-stimulated ATPase activity measurements in cholesterol-loaded Sf9 cell membranes. Although the R147W and R383C variants were expressed in the Sf9 cells, these variants did not show any ABCG2-ATPase activity. The T153M, F373C and S476P variants had lower basal and/or stimulated activities, while the T434M variant showed higher activity than the WT. The stars indicate the results of t-tests: *p < 0.05, **p < 0.01, ***p < 0.001 (at least three independent measurements with three parallels)

In the following experiments, we have transiently expressed the wild-type and the mutant ABCG2 variants in the Sf9 insect cell–baculovirus system, and in Sf9 cell membrane preparations examined the expression levels and the activity of the protein variants (see Fig. 2).

As expected, in this system we could express all the ABCG2 protein variants at similar, high levels, and even the variants not expressed in the human cell lines (R147W and R383C) could be properly expressed (see Fig. 2a—it has been well documented that Sf9 cells can efficiently produce membrane proteins with folding or processing problems as well—[30]).

Figure 2b demonstrates the ABCG2 ATPase activity in this expression system. Basal, prazosin-stimulated, and quercetin-stimulated ABCG2-ATPase activities were measured in the isolated membrane vesicles. In these experiments, equal amounts of membrane proteins were added to the assay and the ABCG2-specific ATPase activity was determined by the addition of a selective ABCG2 inhibitor, Ko143 (see “Materials and methods”). As documented, although showing similar membrane expression levels in the Sf9 cells, the R147W and R383C ABCG2 variants had neither basal nor drug-stimulated activities in these experiments. The ABCG2-T153M, F373C, and S476P proteins had lower basal and/or stimulated ATPase activity, while the T434M variant, interestingly, showed higher basal and prazosin-stimulated activities. The activity of the ABCG2-K360Δ protein was similar to the WT protein.

### Stable expression of the ABCG2 variants in HeLa cells

To further examine the effects of these mutations, we have generated stable HeLa cell lines expressing the ABCG2 variants, by using the Sleeping Beauty transposon-transposase system (see “Materials and methods”). The transposon vector contained the ABCG2-IRES2-EGFP sequence inserted from the vector used in the transient expression studies. Thus, the stable cell lines expressed both the ABCG2 variants and EGFP from the same vector. In order to express the ABCG2 protein at a relatively uniform and low level, better representing the potential physiological expression levels than the transiently expressing HEK cells, the low EGFP-expressing HeLa cells were sorted out for these experiments. To examine the *ABCG2* gene copy numbers in the low-expressing cells, a qPCR assay was performed in the gDNA isolates. The results shown in Suppl Fig. 1 demonstrate that the vector was inserted only in low copy numbers in the stable cell lines, the *ABCG2* copy numbers showing values between 1 and 3.

**Fig. 3:**
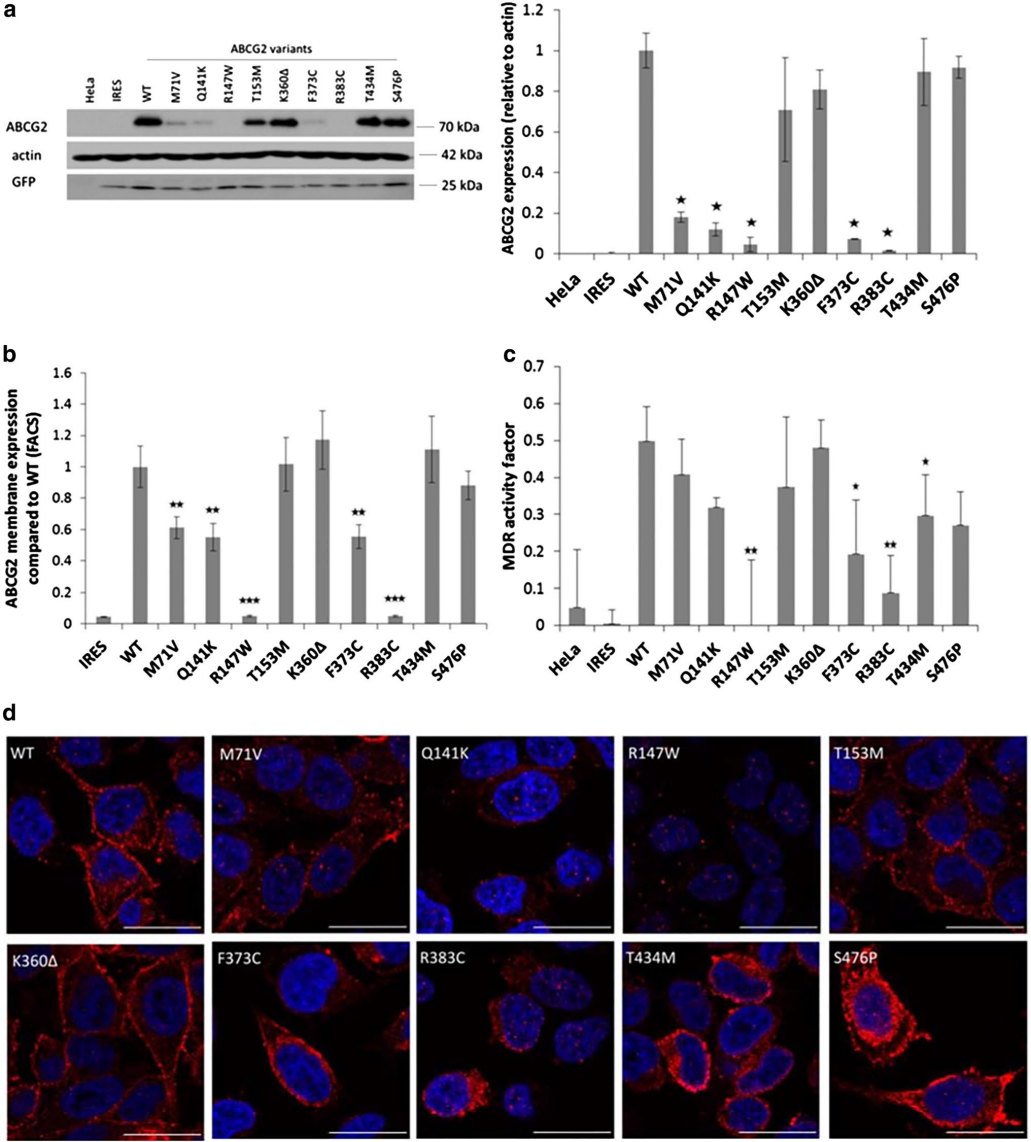
ABCG2 expression and dye extrusion capacity measurements in stable HeLa cell lines, expressing the ABCG2 variants. The cell lines were generated by a transposon-based system and selected to express low levels of the protein. **a** Western blot analysis of stable HeLa cells (left panel: representative Western-blot, right panel: densitometry results of three blots). The ABCG2 M71V, Q141K, R147W, F373C and R383C variants showed low levels of ABCG2 proteins in these cells. **b** Membrane expression of the mutant proteins. The R147W and R383C variants did not reach the plasma membrane. We found lower plasma membrane expression levels in the case of the M71V, Q141K and F373C variants (four independent experiments). **c** Hoechst dye extrusion was not measurable in the case of the R147W and R383C variants, while lower dye extrusion capacity was observed in the case of the F373C and T434M variants (four independent experiments). **d** Representative confocal images of stable HeLa cells. Intracellular localization was observed for the R147W and R383C variants, while the other variants showed mainly plasma membrane expression. The scale bar represents 20 μm. Blue: DAPI, red: ABCG2. The stars indicate the results of t-tests; *p < 0.05, **p < 0.01, ***p < 0.001

Figure 3a demonstrates Western blotting studies for the total protein expression of ABCG2 variants in stable HeLa cell lines. These results show similar expression levels of the K360Δ, T434M, and S476P ABCG2 variants to that of the WT protein. Somewhat lower expression was found for the ABCG2-T153M protein, and significantly lower stable expression levels were observed for the M71V, Q141K, and F373C variants. Practically no stable protein expression was seen for the R147W and R383C ABCG2 variants. These results show the potential for a more sensitive discrimination of the genetic variants in the stable ABCG2 expression system than that observed in transient expression (see Fig. 1a).

In order to clarify the potential sources of the differences in protein expression of the ABCG2 variants in the stable HeLa cell lines, we have determined the respective mRNA levels in these cells. According to the qPCR studies, similar ABCG2 mRNA expression levels were found in all the stable cell lines expressing the ABCG2 variants (see Suppl. Figure 2). Thus, differences in the mRNA expression levels may not cause the observed differences in the protein expression of the variants.

In the following experiments, shown in Fig. 3b, we measured the plasma membrane expression levels of the ABCG2 variants in the low EGFP-expressing stable HeLa cells by 5D3 antibody binding, using flow cytometry. These results closely corresponded to the total protein measurements, that is, high membrane expression levels (similar to the WT protein) were observed for the K360Δ, T434M, and S476P ABCG2 variants. In these experiments the ABCG2-T153M protein also had a similar cell surface expression. Significantly lower membrane expression was found for the M71V, Q141K, and the F373C variants, while no membrane expression was observed for the R147W and R383C variants.

In Fig. 3c, we show the results for the Hoechst dye extrusion activity of the ABCG2 variants stably expressed in the HeLa cells. As documented, there was no ABCG2-dependent transport activity in the stable cell lines expressing the R147W and R383C variants; lower transport activity was found for the F373C and T434M variants, while the Hoechst dye extrusion capacity was similar to the WT ABCG2 in cells expressing the M71V, Q141K, T153M, K360Δ, and S476P ABCG2 variants.

Confocal images from these stable HeLa cell lines (Fig. 3d) showed that the R147W and R383C variants localized exclusively intracellularly. Lower expression levels than the wild-type, and partial plasma membrane localization could be detected in the case of the M71V, Q141K, T153M variants. The K360Δ, F373C, T434M, and S476P variants showed similar high expression and membrane localization as the WT ABCG2 protein.

**Fig. 4:**
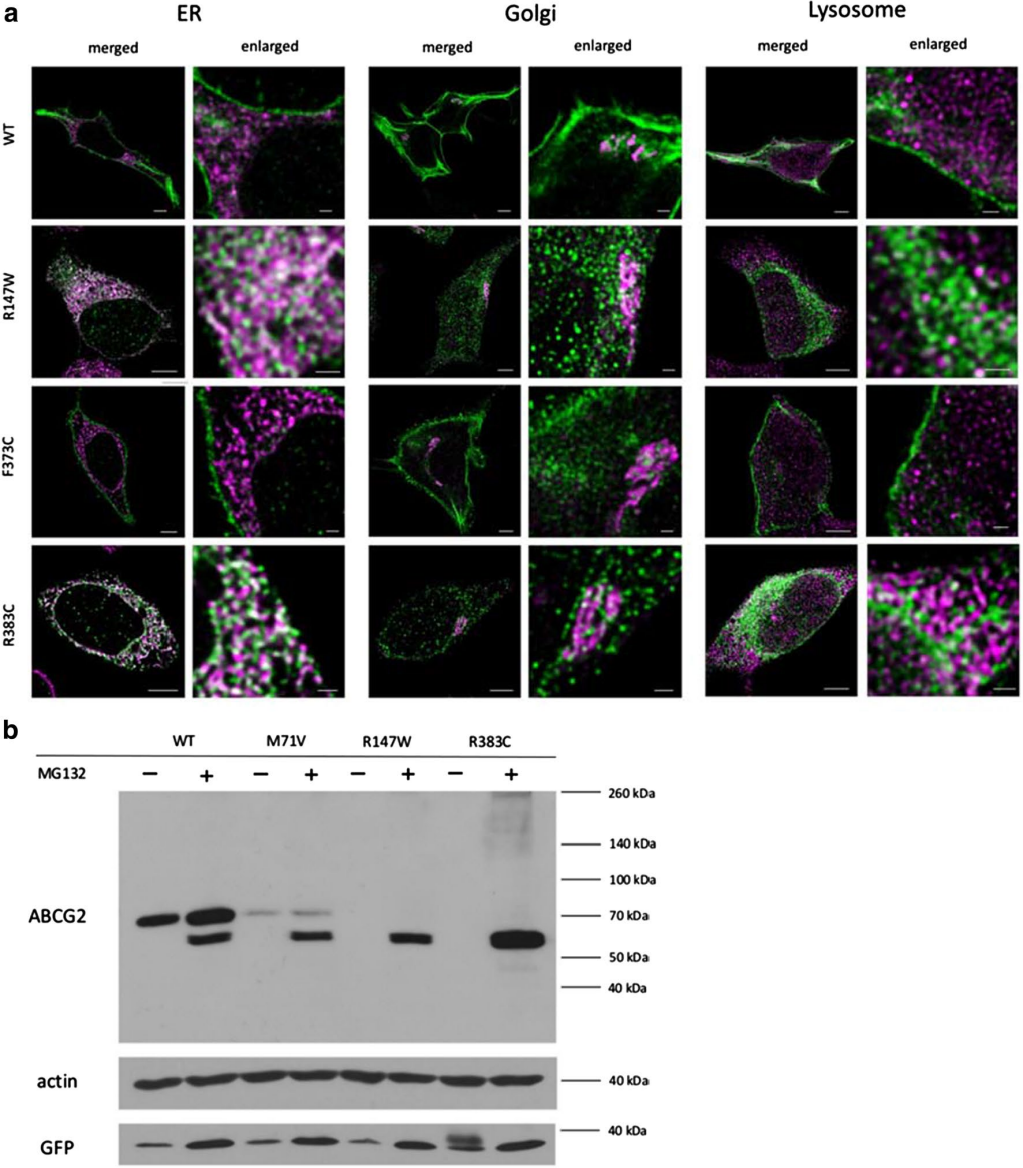
**a** HyVolution 2 pseudo-super-resolution images of HeLa cells stably expressing the wild-type ABCG2 and the R147W, F373C or R383C variants. Co-staining of ABCG2 (green) and the ER marker GRP78 (magenta) showed co-localization in the case of R147W and R383C, indicating that these variants are retained in the ER. Co-staining of ABCG2 (green) and the Golgi marker Giantin (magenta) showed that the WT and F373C ABCG2 proteins partly localize in the Golgi system. Co-staining of ABCG2 (green) and a lysosome marker LAMP1 (magenta) showed only a low level of co-localization in all cases. The scale bars represent 5 μm in the merged and 1 μm in the enlarged pictures. **b** Western blot images of protein samples isolated from HeLa cells stably expressing WT, M71V, R147W and R383C variants. Non-treated (-) and 2 µM MG132-treated cells for 24 hours (+) were examined. Western blot was performed according to the protocol described in the “Methods” section, using antibodies against ABCG2, actin and GFP (see Fig. 1)

In the following experiments we have performed detailed, HyVolution 2 pseudo-super-resolution microscopy studies using the stable cell lines expressing ABCG2 mutant variants showing impaired expression and localization. In these studies, using specific organellar markers, we attempted to find the place of intracellular retention of these ABCG2 variants. As presented in Fig. 4a, we found that the wild-type ABCG2 protein was predominantly localized in the plasma membrane, with some Golgi staining. Most of the ABCG2-R147W and R383C variants, expressed at a low level, localized in the ER compartment. The poorly processed F373C variant was also predominantly localized in the ER, while partly in the Golgi and partly in the plasma membrane compartments. We found no significant co-localization of any of the immunostained ABCG2 variants with lysosomal markers.

In the following experiments, we have studied the effects of the selective and powerful proteasome inhibitor MG132 on the expression of the ABCG2 variants in the stable cell lines. As shown in Fig. 4b, in the case of the WT protein, proteasome inhibition results in an increase in the expression of the fully glycosylated protein (upper band) and the appearance of the non-glycosylated ABCG2 (lower band). This indicates a partial degradation of both the glycosylated and the non-glycosylated WT protein in the proteasomes. In the control cells the fully glycosylated ABCG2-M71V mutant variant shows a much lower level of expression than the WT protein, while in the case of proteasome inhibition, most of this protein appears non-glycosylated. Interestingly, in the case of the ABCG2-R147W and R383C mutant variants there is practically no expression in the control cells, while a major non-glycosylated band appears when proteasome activity is inhibited. This clearly coincides with the ER accumulation of these variants, seen in the co-localization studies (Fig. 4a).

## Discussion

The ABCG2 multidrug transporter protein has several important roles in normal human tissues. In terms of localization, ABCG2 can be found mainly in the tissue interfaces (blood–brain barrier, placenta, liver, intestine), where this transporter performs the removal of numerous endogenous and exogenous harmful substances [1, 31]. Mutations or SNPs in the *ABCG2* gene may influence the transport of these substrates, leading to pathological alterations in the human body. The reduced uric acid excretion by ABCG2 in the intestine leads to hyperuricemia and gout [11]. ABCG2 also modulates the ADME-Tox properties of several drugs; thus an inadequate functioning of ABCG2 may dramatically change the side effects of these compounds [8].

ABCG2 has been reported to be present in several naturally occurring variants in the human populations. Polymorphisms in the ABCG2 gene are frequent in Japan and in other Eastern countries [32–34], where the most studied variants are the Q141K (MAF: 0.16–0.32), and Q126X (MAF: 0.001), resulting in reduced-function or non-functional protein expression. In Caucasian populations, the Q126X variant has not been found, while, in addition to the frequently occurring Q141K variant (MAF: about 0.1), the R236X (truncated, non-functional variant) and the M71V, Q141K, R147W, T153M, K360Δ, F373C, R383C, T421A, T434M, and S476P ABCG2 variants were also reported to occur in several individuals [20, 22, 35, 36].

The most common variant, Q141K, has been described to increase the risk of hyperuricemia and gout; moreover, it has a significant effect on earlier onset of gout and was found to be associated with family history of the disease [36]. Recently it has been described that ABCG2 dysfunction is a strong independent risk factor for pediatric-onset hyperuricemia/gout [35].

It should be mentioned that the second most frequent variant of the ABCG2 is V12M. As we have previously reported, V12M does not alter the expression of the protein in the red blood cell membranes of the carriers [19]. It has also been demonstrated that this variant does not affect the urate transport activity of the protein [34], while data from high-throughput studies [37, 38] and meta-analysis of several published results [36] indicated that this SNP may be in fact protective against gout.

In the present study we have performed a comprehensive, detailed study of naturally occurring ABCG2 variants. A total of nine previously identified ABCG2 variants were tested—the M71V, Q141K, R147W, T153M, K360Δ, F373C, R383C, T434M, and S476P variants were expressed and analyzed in several assay systems. We have devised a suitable (EGFP corrected) transient mammalian cell expression systems for the evaluation of the ABCG2 variants, allowing a relatively rapid and simple determination of both protein expression and function. We have also applied an Sf9 insect cell—baculovirus expression system, in which membrane protein expression is hardly affected by folding or trafficking problems, to directly study ABCG2 function. In addition, a transposon-based stable mammalian cell expression system, also correctable for EGFP expression, was applied to perform detailed studies on protein folding, trafficking, membrane localization, and transport activity. In these stable cell lines ABCG2 and EGFP are expressed from the same vector and, as the cDNAs are separated by an IRES sequence, EGFP expression does not influence the expression of ABCG2. By sorting out cells with low EGFP expression, we could generate stable HeLa cell lines with low inserted copies of *ABCG2,* closely corresponding to physiological ABCG2 expression. In addition to the present studies, these cell lines are useful for a detailed examination of the effects of various drugs, potentially modifying the expression, trafficking or function of the naturally occurring variants of ABCG2.

Based on the data presented in the results section, we conclude that the R147W and R383C ABCG2 protein variants are seriously damaged, practically not expressed in the mammalian cells. The high-resolution confocal microscopy results indicate that the small amounts of these ABCG2 variants do not reach the plasma membrane, are localized mainly in the ER, and thus have major folding problems. The M71V, Q141K, F373C, and T153M ABCG2 variants are expressed in variable amounts in the mammalian cells but are found only at low levels in the plasma membrane. As studied in detail, the F373C variant is mostly localized in the Golgi and other intracellular compartments, while we found no apparent accumulation of any of the ABCG2 variants in the lysosomes. Previous studies have shown that the expression of the frequent ABCG2-Q141K variant is altered at several levels—reduced mRNA maturation, protein folding, membrane trafficking, and increased degradation of this protein have been suggested [24–27]. For the M71V variant our group demonstrated a reduced folding and trafficking in mammalian cells [21], while the partially defective F373C and T153M variants have not been studied as yet at the cellular level.

Since immunostaining does not recognize a partially degraded protein in the lysosomes, we have explored the effects of the selective proteasome inhibitor MG132 on the cellular fate of the ABCG2 variants (see Fig. 4b). We found that in the case of the WT protein, proteasome inhibition results in an increase in the expression of the fully glycosylated protein, and the presence of a small amount of the non-glycosylated ABCG2, normally eliminated by the proteasomes. In contrast, in the case of the ABCG2-R147W and R383C mutant variants, there is practically no expression of the fully glycosylated protein, while the appearance of a major non-glycosylated band when the proteasome activity is inhibited indicates that the misfolded, non-glycosylated protein (retained in the ER) is eliminated by the proteasomes. The ABCG2-M71V mutant variant shows lower expression than the WT protein, and proteasome inhibition results in the appearance of a large amount of the non-glycosylated protein, indicating partial ER retention and proteasomal degradation of this variant. These experiments indicate that the WT ABCG2 goes through a cellular processing with only a minor portion retained in the ER and degraded by the proteasomes, while the variants variably mis-folded are mostly retained in the ER and rapidly eliminated by the proteasomes.

An important finding in the present work is that the K360Δ, T434M, and S476P ABCG2 variants are properly expressed and functional in the plasma membrane and thus, in spite of being missense variants, may not cause any pathological alterations. In the case of the ABCG2-T434M protein, the Hoechst dye extrusion experiments showed similar or lower transport capacity, while in the ATPase experiments, we measured a higher activity. These findings may indicate a slight functional and/or trafficking problem with this variant.

While this manuscript was under review, Toyoda et al. [23] reported an analysis of the variants R147W, T153M, K360Δ, F373C, T421A, T434M, S476P, S572R, D620N at the clinical level and performed a detailed cellular study using HEK cells, membrane vesicles, and a Xenopus oocyte expression system. The expression and localization of these variants, as reported in this paper, in most cases are in accordance with our current results and clearly establish the potential clinical role of the non-functional variants. Still, the approaches were somewhat different, e.g. our stable cell lines, expressing low levels of an untagged ABCG2, may be more informative regarding mild changes in expression and trafficking, as the GFP tag on the ABCG2 protein may alter these parameters (see in [21], Suppl. Figure 5). A potential result of these differences is that Toyoda et al. [23] concluded that the T434M and S476P variants (expressed with a GFP tag) did not have full activity in urate transport experiments. In contrast, we could not observe such differences in our transport and ATPase experiments with the untagged protein (using several well-established substrates of the ABCG2 protein, including Hoechst 33342, quercetin and prazosin).

**Fig. 5:**
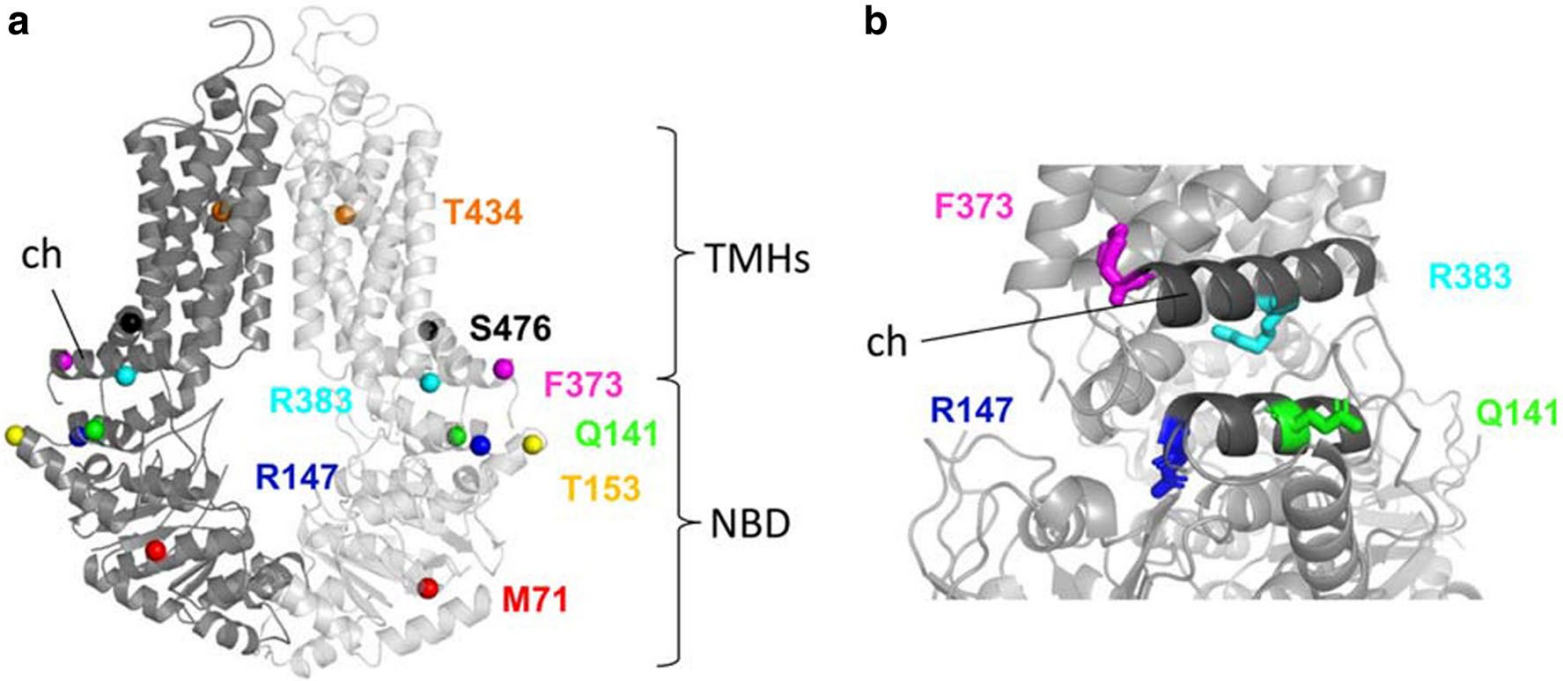
**a** Localization of the studied missense variants in the structure of the ABCG2 protein (based on Jackson et al. [28]). **b** The seriously damaged R147W, R383C variants and the Q141K, F373C variants—which showed significantly lower expression and activity in our experiments—localize in the so-called connector area in the ABCG2 structure (dark grey), demonstrating a key role of this region in the expression, maturation and activity of the ABCG2 protein. TMHs: transmembrane helixes, NBD: nucleotide-binding domain, ch: connector/elbow helix

In Fig. 5a, the positions of the studied amino acid variants are depicted in the recently published atomic level model of the human ABCG2 protein [28]. The two non-expressing mutant variants (R147W and R383C), and two of the variants which showed lower expression (Q141K, F373C) are localized to the so-called connector region (see Fig. 5b). This region has been shown to be crucially important in the stabilization and the structural rearrangements during the ABCG2 transport cycle [26, 39], and an artificial mutation in the R383 position (R383A) was reported to damage the function and trafficking of this variant [40]. All these data further emphasize the key role of this region within the ABCG2 protein and predict a damaging effect of mutations in this region. As shown by us previously [21], the M71V variant, with reduced expression and trafficking, is localized in the NBD, and may directly alter the folding and maturation of the protein.

An interesting ABCG2 variant is the K360Δ protein, in which a lysine is deleted in a flexible intracellular loop, which is actually not seen in any of the atomic level models [28, 41–43]. This lysine is close to other lysines (K357, K358), previously identified as ubiquitination sites of the ABCG2 protein [44, 45]. The high expression of this variant in some of our experiments may suggest that the ubiquitin-mediated degradation of this variant is reduced.

In summary, the studies presented in this paper indicate that two naturally occurring ABCG2 variants, R147W and R383C, are detrimental to both expression and function, and thus heterozygous and especially homozygous or compound heterozygous patients carrying these ABCG2 variants may be especially susceptible to gout formation and drug toxicity, while less likely to have drug resistant tumors (in addition they may show a Junior-blood group feature—see [46]). Moreover, any potential treatment promoting the trafficking of these variants to the plasma membrane will not correct the defective function of these variants.

Individuals carrying the M71V, Q141K, T153M, and F373C ABCG2 variants, especially in a compound heterozygous form, may have similar phenotypic and pathological features, which is less efficient trafficking and function. In the case of these individual variants, a therapy promoting trafficking to the plasma membrane may significantly improve ABCG2 function. The expression of T434M and S476P proteins are not damaged; however, they may show somewhat altered transport function. In contrast, the ABCG2-K360Δ is a fully functional variant, with a potential of increased plasma membrane expression.

While the present experiments help to clarify the cellular phenotype of the specific ABCG2 variants examined, the transient expression system (yielding parallel ABCG2 and EGFP expression) used in this study provides a relatively simple and rapid screening tool. This system can be used for assessing the cellular expression, thus it allows to estimate potential medical effects (e.g. drug sensitivity or susceptibility to gout) in individuals carrying these mutations. The results presented here may also help to elucidate the specific role of the affected regions in the folding and functioning of the ABCG2 protein.

## Materials and methods

### Vector constructs

pIRES2-EGFP vector was a kind gift from László Nyitray (ELTE Biochemistry Department). We have created the constructs containing the wild-type (WT), M71V, and Q141K ABCG2 variants in our previous work [21]. To generate the other mutant variants, two-step PCR mutagenesis was performed, using the WT ABCG2-containing pIRES2 vector template amplified by primer pairs containing the desired mutation (see Suppl. Figure 3a). The ABCG2-IRES2-EGFP cassette was inserted in the pIRES2-EGFP vector after digestion by BamHI and NheI enzymes (for vector map see Suppl. Figure 4a).

The original transposon-transposase vectors were generated previously by Tamas Orban’s group (see [47], correction: [48]). The mutant variants of the ABCG2-IRES2-EGFP cassette were inserted between IR-DRs in the transposon vector by using the NheI and AgeI enzymes, after PCR amplification with a backbone-specific reverse and an overhang forward primer that contained the desired AgeI cleavage sequence (see Suppl. Figures 3b, 4b). In addition to the mutant ABCG2 variant-containing vectors, we have generated a vector with the IRES2-EGFP cassette without the ABCG2 sequence, to serve as a control in these experiments.

For Sf9-baculovirus expression studies we cloned the mutant versions of *ABCG2* cDNA from pIRES2 vectors with NotI/SacI into the pAcUW-21-L plasmid (vector was a gift from Csilla Laczka-Ozvegy) after PCR amplification with primers containing restriction enzyme cutting site overhang (see Suppl. Figure 3c).

### Mammalian cell culture, transfection, and MG132 treatment

HEK293H and HeLa cells were grown in DMEM/high glucose/GlutaMAX medium (Gibco, cat. 10569010) completed with 10% FBS (Gibco, cat. 1640071) and 1% penicillin–streptomycin (Gibco, cat. 15070063) at 37 °C (5% CO_2_). Transient transfection of HEK293 cells was carried out with lipofectamine 2000 (Invitrogen, cat. 11668019) in Opti-MEM medium (Gibco, cat. 31985070), according to the manufacturer’s protocol. Protein determination and Hoechst dye extrusion measurements were carried out 48 h after transfection. MG132 treatment was performed by culturing the cells for 24 h with 2 µM final MG132 concentration in DMEM/high glucose/GlutaMAX medium. We used a 10 mM MG132 in DMSO stock solution (Sigma-Aldrich, cat. M7449) and an equal volume of DMSO was added to the medium in the case of the non-MG132-treated control cells.

### Generation of stable cell lines

The ABCG2-expressing stable cell lines were generated using the Sleeping Beauty transposon-transposase system. We used a 1:10 transposase coding plasmid: transposon plasmid ratio for transfection. HeLa cells were transfected with the mixed plasmids using Lipofectamine 2000 (Invitrogen, cat. 11668019) in Opti-MEM (Gibco, cat. 31985070). Three days after transfection, the EGFP positive cells were sorted using BD FACS Aria II and seeded on 6-well plates. After two more weeks of culturing, two cell populations were detected among the EGFP positive cells: a population with lower and one with higher EGFP fluorescence. The two separable populations were present, regardless of which mutant variant of ABCG2 they expressed. The low EGFP-expressing cells were sorted and seeded on 6-well plates. Our further experiments were performed using these low EGFP-expressing stable cell lines.

### gDNA isolation and copy number determination

Genomic DNA was isolated from stable cell lines with Gentra Puregene Blood Kit (Qiagen), according to the manufacturer’s protocol for mammalian cell lines. Copy numbers were determined by TaqMan based qPCR as described previously ([47], corr.: [48]). Briefly, the qPCR probes were designed to recognize the left arm (IR-DR) of the integrated Sleeping Beauty cassette. For control copy number determination the *RPPH1* gene was used.

### Hoechst 33342 uptake measurements

Hoechst 33342 (Hst, Thermo Fisher, cat. H1399) uptake was determined either in transiently transfected HEK293 cells (with pIRES2 vectors, Suppl. Figure 4a) 48 h after transfection, or in the stable HeLa cell lines. Cells were trypsinized and then preincubated for 5 min at 37 °C with or without the selective ABCG2 inhibitor, Ko143 (2 µM). Measurements were carried out in FACS Canto II, with continuous sampling for 80 s (linear phase of the uptake). The EGFP-positive cells were gated at 20-s intervals. Initial slopes were calculated from these five points (0–80 s) by linear fitting (according to [21]). MAF (MDR activity factor) values were calculated as described previously [29].

### Plasma membrane expression studies, flow cytometry

We determined the cell surface expression of ABCG2 in the stable HeLa cell lines and in transiently transfected HEK293 cells 48 h after transfection. Antibody labeling was performed in trypsinized cells with the ABCG2-specific 5D3 mouse monoclonal antibody (gift of Bryan Sorrentino, Division of Experimental Hematology, Department of Hematology/Oncology, St. Jude Children’s Research Hospital). Ko143 is an ABCG2 inhibitor that has an impact on ABCG2 conformation, thus helping 5D3 antibody recognition. 1 µM Ko143 (Sigma-Aldrich, cat. K2144) was added to the samples before the measurements. Alexa Fluor 647-labeled IgG2b (Thermo Fisher, cat. A-21242) was used as a secondary antibody. Propidium iodide (final concentration: 1.6 µg/ml, Sigma Aldrich, cat. 4170) was used for dead cell separation. Measurements were carried out in FACS Canto II after gating for live, EGFP positive cells.

### Western blotting

Total protein from the cells was extracted by the addition of TE sample buffer (0.1 M TRIS-PO_4_, 4% SDS, 4 mM Na-EDTA, 40% glycerol, 0.04% bromophenol blue, and 0.04% β-mercaptoethanol; materials from Sigma-Aldrich). Equal amounts of the protein samples were loaded on 10% acrylamide gels. Blots were probed with the following primary antibodies: anti-ABCG2 (BXP-21, Abcam, cat. ab3380), anti-EGFP (Abcam, cat. ab290), and anti-β-actin (Sigma, cat. A1978). Goat anti-mouse IgG (H + L) HRP conjugate (Abcam, cat. ab97023) and goat anti-rabbit IgG (H + L) HRP conjugate (Abcam, cat. ab6721) secondary antibodies were used to visualize and quantify the results. Detection was performed with Clarity Western ECL Substrate (BioRad, cat. 1705060) and luminography. Densitometric analysis was performed by ImageJ software v1.42q.

### Immunostaining

HEK cells were seeded onto 8-well Nunc Lab-Tek II chambered (Thermo Fisher, cat. 155409) at 5 × 10^4^ cells/well density and grown for 48 h after transfection. Stably expressing HeLa cells were seeded onto ibiTreat µ-Slide 8 Well (Ibidi, cat. 80826) at 2 × 10^4^ cells/well density and grown for 24 h. The samples were gently washed with Dulbecco’s modified phosphate-buffered saline (DPBS) and then fixed with 4% paraformaldehyde in DPBS for 10 min at room temperature. After several washing steps, the cells were blocked for 1 h at room temperature in DPBS containing 2% bovine serum albumin, 1% fish gelatin, 0.1% Triton-X 100, and 5% goat serum (blocking buffer). The samples were then incubated for overnight at 4 °C with the primary antibody (Bxp-21, 1:200, Abcam, cat. ab3380) diluted in blocking buffer. After washing with DPBS, the cells were incubated for 1 h at room temperature with the secondary antibody (Alexa Fluor 647-conjugated goat anti-mouse, Thermo Fisher, cat. A-21235) diluted 1:250 in blocking buffer. Nuclei were stained with DAPI (1 µM final concentration). Cells were examined with Zeiss LSM 5710 laser scanning fluorescence confocal microscope (40 ×, oil immersion objective), and images were processed with ZEN 2012 software.

### Immunostaining for pseudo-super-resolution microscopy

The four selected stably expressing HeLa cells (variants WT, R147W, R383C, F373C) were seeded onto ibiTreat µ-Slide 8 Well (Ibidi, cat. 80826) at 2 × 10^4^ cells/well density and grown for 24 h. The samples were gently washed with Dulbecco’s modified phosphate-buffered saline (DPBS) and then fixed with 4% paraformaldehyde in DPBS for 15 min at room temperature. After several washing steps, the cells were blocked for 1 h at room temperature in DPBS containing 2% bovine serum albumin, 1% fish gelatin, 0.1% Triton-X 100, and 5% goat serum (blocking buffer). The samples were then incubated for overnight at 4 °C with the primary antibodies (Bxp-21, 1:200, Abcam, cat. ab3380; Anti-GRP78, 1:500, Abcam, cat. ab21685; Anti-Giantin, 1:1000, BioLegend cat. 924302; Anti-LAMP1, 1:250 AntibodyGenie cat. CAB2582) diluted in blocking buffer. After washing with DPBS, the cells were incubated for 1 h at room temperature with the secondary antibodies (Alexa Fluor 568 conjugated goat anti-mouse, Thermo Fisher, cat. A-11004; Alexa Fluor 647-conjugated goat anti-rabbit, Thermo Fisher, cat. A-21244), diluted 1:250 in blocking buffer. The samples were examined with Leica HyVolution 2 pseudo-super-resolution imaging method, using a Leica TCS SP8 STED microscope equipped with a Leica HC PL APO 100 × STED white (1.4 NA) objective. The Alexa Fluor 568 and 647 dyes were excited with 552 and 638 nm laser, respectively. The fluorescence of each dye was detected sequentially and spectrally by a hybrid detector at 590–630 nm and 650–700 nm wavelengths, respectively. The pinhole diameter was set to 0.6 AU in each channel. Huygens Pro deconvolution software was used for image restoration and Leica LAS X 3.1.1 software was used for image analysis.

### Expression in Sf9 cells, membrane preparation, ATPase measurements

Sf9 insect cells were grown in suspension at 27 °C in TNM-FH medium (Sigma-Aldrich, cat. T3285) supplemented with 5% FBS. The cells were transfected with flashback ULTRA Kit (Oxford Expression Technologies Ltd.) according to the manufacturer’s protocol. Membrane expression studies were carried out as described previously [49].

ATPase activity of WT ABCG2 and the mutant variants was measured by colorimetric detection of inorganic phosphate liberation in microplates, as described previously [50, 51]. Protein concentrations (5 μg/well) were normalized according to Western blots for ABCG2; thus equal amounts of the ABCG2 variants were used in the measurements. Cholesterol loading of the membranes was achieved by the addition of 0.6 mM RAMEB-cholesterol (Cyclolab Ltd, Budapest, Hungary) before measurements, and the membranes incubated for 10 min on ice, according to Telbisz et al. [49]. The measurement of the ATPase activity was started with the addition of 3.1 mM MgATP, the samples incubated at 37 °C, and the reaction terminated after 25 min with the P-reagent [51]. Photometric measurements were carried out in Victor Multilabel plate reader (PerkinElmer) at 660 nm. ABCG2-specific ATPase reaction was verified by adding the ABCG2-specific inhibitor Ko143 to the reaction. We used the following concentration of ABCG2 inhibitors and substrates: vanadate 1 mM, Ko143 1 μM, quercetin 5 μM, prazosin 20 μM.

### mRNA level measurements

mRNA was purified from the stable, ABCG2-expressing HeLa cells with PureLink MiniKit (Thermo Fisher Scientific, cat. 12183018A) according to the manufacturer’s instructions. After cDNA conversion (Thermo Fisher Scientific, High capacity cDNA reverse transcription kit, cat. 4368814) mRNA levels were determined with qPCR probes against ABCG2 (Thermo Fisher Scientific, cat. 00184979) and RPLP0 (cat. 99999902), with TaqMan Universal Master Mix (Thermo Fisher Scientific, cat. 4304437) according to the manufacturer’s protocol. Reactions were run on a StepOnePlus™ platform (Thermo Fisher Scientific), and ABCG2 mRNA levels were normalized with ΔΔ*C*t method to RPLP0 mRNA levels.

### Electronic supplementary material

Below is the link to the electronic supplementary material.

Supplementary material 1 (PDF 393 kb)
