# Supplementary material for: Cellular expression and function of naturally occurring variants of the human ABCG2 multidrug transporter
Source: Cell Mol Life Sci. 2019 Jun 28;77(2):365–78. doi: 10.1007/s00018-019-03186-2 (PMC6971004; doi:10.1007/s00018-019-03186-2)

Supplementary Materials for the paper

'Cellular expression and function of naturally occurring missense variants of the human ABCG2 multidrug transporter'

Suppl. Fig. 1 Copy number determination of stable HeLa cell lines expressing the different variants of ABCG2. Comparative ΔΔCt method, see further details in Methods.

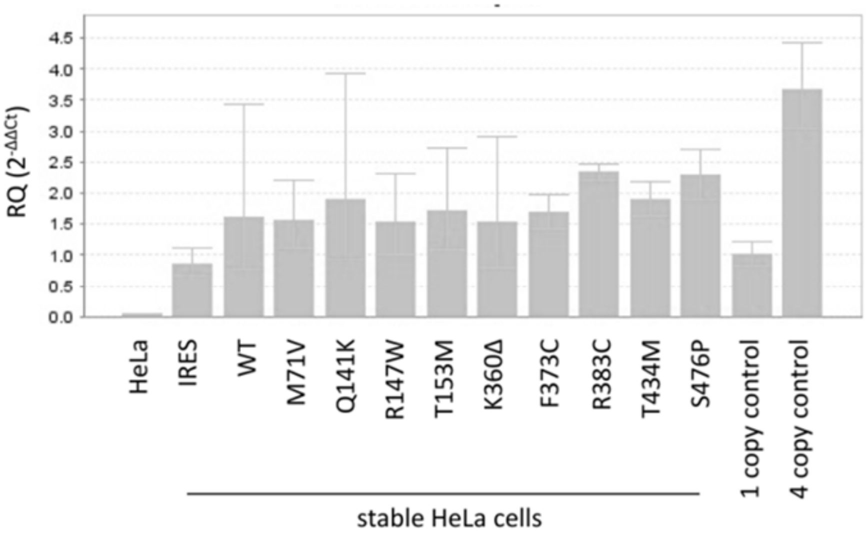

Suppl. Fig. 2 mRNA expression of WT ABCG2 and variants in stable HeLa cells. **A.** Gel image of isolated RNAs. **B.** The results of qPCR reactions determining RNA expression. Comparative ΔΔCt method, the probes used: ABCG2, between 5-6 exon (00184979) and RPLP0, control (99999902).

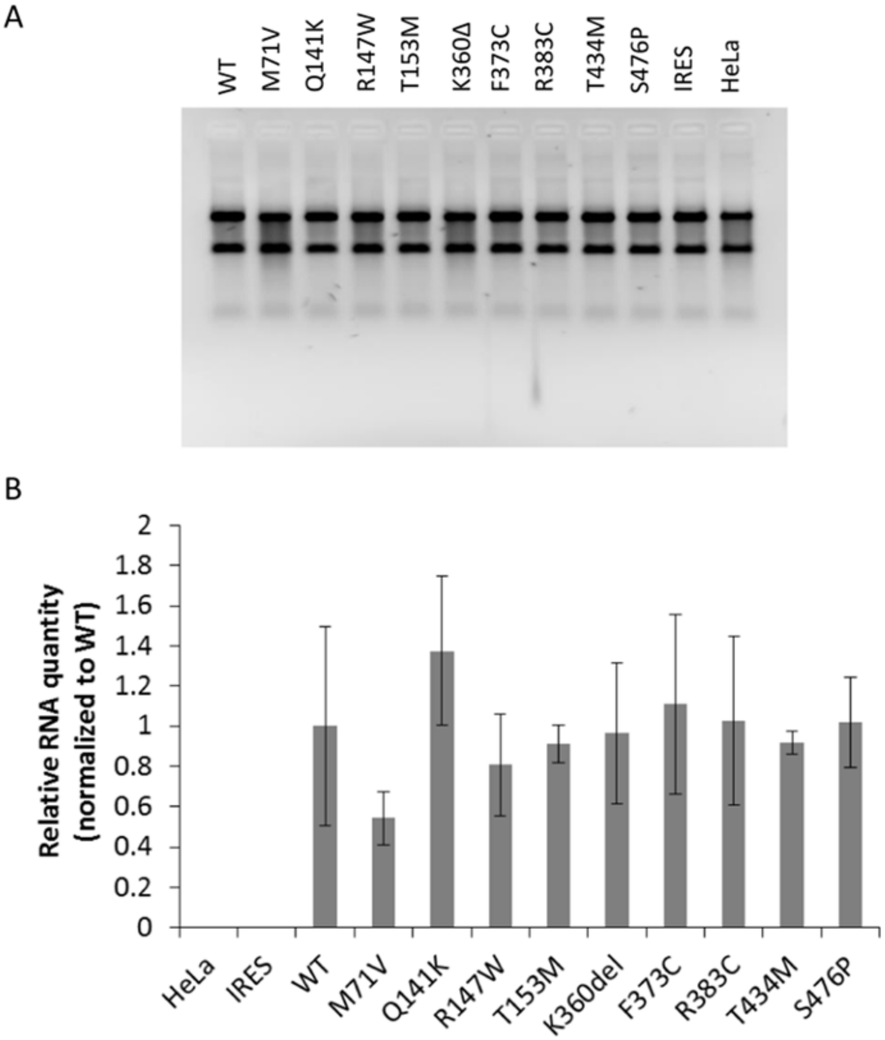
A
WT
M71V
Q141K
R147W
T153M
K360Δ
F373C
R383C
T434M
S476P
IRES
HeLa
B
Relative RNA quantity
(normalized to WT)
2
1.8
1.6
1.4
1.2
1
0.8
0.6
0.4
0.2
0
HeLa
IRES
WT
M71V
Q141K
R147W
T153M
K360del
F373C
R383C
T434M
S476P

Suppl. Fig. 3. Primers used **A.** Mutagenesis primers. **B.** Primers used in subcloning into p10-SB vector. **c** Primers used in subcloning to pAcUW Sf9 expression vectors.

A

Mutagenesis primers

R147W (rs372192400) CGG>TGG

F: 5' GTTCTCAGCAGCTCTTTGGCTTGCAACAACTATG 3'

R: 5' CATAGTTGTTGCAAGCCAAAGAGCTGCTGAGAAC 3'

T153M (rs753759474) ACG>ATG

F: 5' CGGCTTGCAACAACTATGATGAATCATGAAAAAAACGAAC 3'

R: 5' GTTCGTTTTTTTCATGATTCATCATAGTTGTTGCAAGCCG 3'

F373C (rs752626614)

F: 5' CTTCAAGGAGATCAGCTACACCACCTCCTGCTGTCATCAACTCAGATG 3’

R: 5' GAAACCCATCTGAGTTGATGACAGCAGGAGGTGGTGTAGCTGATC 3'

R383C (rs560659849) CGT>TGT

F: 5' CTCAGATGGGTTTCTAAGTGTTCATTCAAAAACTTGCTGG 3'

R: 5' CCAGCAAGTTTTTGAATGAACACTTAGAAACCCATCTGAG 3'

T434M (rs769734146) ACG>ATG

F: 5' GTTCTCTTCTTCCTGATGACCAACCAGTGTTTC 3'

R: 5' GAAACACTGGTTGGTCATCAGGAAGAAGAGAAC 3'

S476P (-) TCT>CCT

F: 5' CCTTGGAAAACTGTTACCTGATTTATTACC 3'

R: 5' GGTAATAAATCAGGTAACAGTTTTCCAAGG 3'

K360del (rs750972998) TCT>-

F: 5' GTGAGAAGAAGAAGATCACAGTCTTCAAGG 3'

R: 5' CCTTGAAGACTGTGATCTTCTTCTTCTCAC 3'

Vector backbone and insert specific primers used with the mutagenesis primers:

CMV for

5' TAGGCGTGTACGGTGGG 3'

IRES2 rev

5' TATAGACAAACGCACACCG 3'

B

Primers used for insertion of ABCG2 cDNAs into SB-p10 vector:

CMV for

5' TAGGCGTGTACGGTGGG 3'

G2-AgeI rev

5' AGTGCAGACCGGTCGGATCCAATTTAAGAATATTTTTTAAG 3'

C

Sf9 primers:

NotI-G2-for: 5’ ATCAGCGGCCGCATGTCTTCCAGTAATGTCGAAG 3’

SacI-G2-rev: 5’ ATCTGAGCTCTTAAGAATATTTTTTAAGAAATAACAATTTC 3’

Suppl. Fig. 4. A. Plasmid used in transient expression experiments. **B.** Sleeping Beauty plasmid used in the generation of stable HeLa cell lines.

A

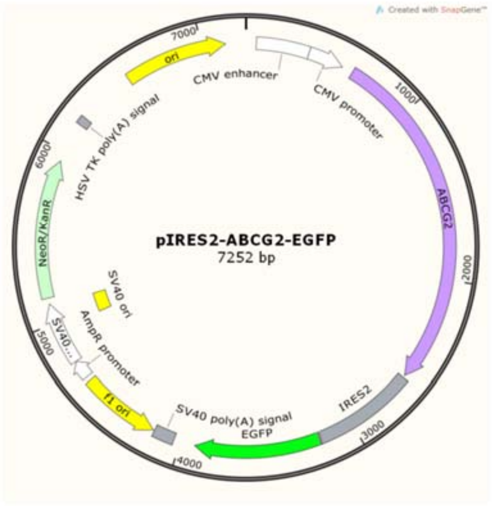

B

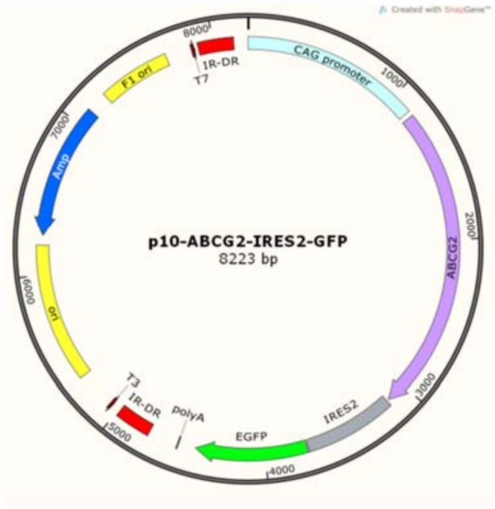

Supplement: Supplementary file 1 — Supplementary material 1 (PDF 393 kb) [file 18_2019_3186_MOESM1_ESM.pdf]
